# Relationship between White Blood Count to Mean Platelet Volume Ratio and Clinical Outcomes and Severity of Coronary Artery Disease in Patients Undergoing Primary Percutaneous Coronary Intervention

**DOI:** 10.1155/2020/9625181

**Published:** 2020-08-13

**Authors:** Altekin Refik Emre, Kilinc Ali Yasar, Yanikoglu Atakan, Cicekcibasi Orhan, Kucuk Murathan

**Affiliations:** ^1^Department of Cardiology, Faculty of Medicine, Akdeniz University, Antalya, Turkey; ^2^Department of Cardiology, Akcakoca State Hospital, Akcakoca, Duzce, Turkey; ^3^Department of Cardiology, Private OFM Antalya Hospital, Antalya, Turkey

## Abstract

**Background:**

The white blood cell count to mean platelet volume ratio (WMR) is an indicator of inflammation in patients with atherosclerotic disease. Residual SYNTAX Score (RSS) is an objective measure of degree and complexity of residual stenosis after percutaneous coronary intervention (PCI). We investigated the relationship between WMR and clinical prognosis and RSS in patients undergoing primary percutaneous coronary intervention (P-PCI).

**Method:**

Between June 2015 and December 2018, 537 patients who underwent primary PCI were evaluated for in-hospital events, and 477 patients were evaluated for clinical events during follow-up after discharge. The endpoint of our study is major adverse cardiac events (MACEs) seen in the in-hospital and follow-up periods.

**Results:**

In our study, 537 patients were stratified into two groups according to admission median WMR. There were 268 patients in the low WMR group (WMR < 1286) and 269 patients in the high WMR group (WMR ≥ 1286). RSS (*p* = 0.01) value of the high WMR group was higher than that of the low WMR group. The rates of in-hospital MACE (*p* = 0.001), cardiac death (*p* < 0.001), decompansated heart failure (0.007), and ventricular tachycardia/fibrillation (*p* = 0.003) were higher in the high WMR group than in the low WMR group. The follow-up MACEs (*p* = 0.043), cardiac death (*p* = 0.026), and reinfarction (*p* = 0.031) ratio were higher in the high WMR group. In ROC analysis, cut-off values of in-hospital and follow-up MACEs were >1064 (sensitivity: 83.12%, and specificity: 36.29%) and >1130 (sensitivity: 69.15%, and specificity: 44.91%), respectively. The Kaplan-Meier analysis showed that the high WMR group had the significantly lowest MACE-free survival rate (log-rank test, *p* = 0.006). A moderate correlation was observed between WMR and RSS (*r*: 456, *p* = 0.002).

**Conclusion:**

A higher WMR value on admission was associated with worse outcomes in patients with P-PCI and independently predicted for follow-up MACEs. The WMR provides both a rapid and an easily obtainable parameter to identify reliably high-risk patients who underwent primary percutaneous coronary intervention due to STEMI.

## 1. Introduction

Atherosclerotic plaques rupture and contribute to thrombus formation by inflammatory mechanisms, which can lead to the development of ST elevation myocardial infarction (STEMI). White blood cells (WBCs) and platelets have potential roles in the pathogenesis of STEMI. WBCs play a crucial role in the progression of atherosclerosis and destabilization and rupture of a plaque, leading to thrombotic events. Previous studies have reported increased admission WBC count to be a robust predictor of morbidity and mortality in patients with acute coronary syndrome (ACS) [1, 2]. Platelets play a key role in atherothrombosis leading to ACS, and patients with increased platelet activation are at higher risk of cardiovascular events in the setting of ACS. Mean platelet volume (MPV) is a highly sensitive marker of platelet activity, and it could link the pathophysiology of diseases related to thrombosis and inflammation [3, 4].

STEMI patients may have a non-negligible mortality risk and relatively high long-term incidence of cardiac events. Effective risk classification is important for STEMI patients in terms of treatment planning and prognosis. Although clinical risk scores such as GRACE and TIMI are used for this purpose in daily practice, they do not include inflammation parameters [5]. For this reason, there is a high demand for a reliable, accessible, noninvasive, and hematological prognostic marker in ACS, which would identify patients of high cardiovascular risk in secondary prevention and tailor the therapy to their needs. As the understanding of the role of inflammation in the atherosclerotic process gets better, studies have focused on new inflammatory hematological indices for improved evaluation of the risk [6]. The main advantage of hematological indices is that they are relatively inexpensive and thus widely and easily available in daily clinical practice. WBCs, MPV, platelet-to-lymphocyte ratio (PLR), and neutrophil-to-lymphocyte ratio (NLR) are some of the inflammatory markers that have been demonstrated to have predictive and prognostic significance in cardiovascular diseases [7, 8]. WBC count to MPV ratio (WMR) can be obtained from routine blood tests and used as a cost-effective new biomarker for inflammation. The WMR is increasingly gaining importance as a promising prognostic marker in atherosclerotic disease [9, 10].

At the time of primary percutaneous coronary intervention (P-PCI), 40–65% of the patients exhibit one or more concomitant coronary lesions (i.e., multivessel disease (MVD)). The presence of narrowed coronaries other than those related to index ischemia in patients with STEMI is suggested as a feature associated with adverse clinical outcomes [11]. The Residual SYNTAX Score (RSS) was developed to assess quantitatively the degree and complexity of residual stenoses, based on recalculating the SYNTAX Score (SS) from coronary angiography after PCI. Higher RSS has been associated with worse outcomes in patients undergoing angiography-mediated PCI [12]. In a post hoc analysis of the ACUITY trial including patients with moderately high-risk non-ST elevation myocardial infarction (non-STEMI) acute coronary syndromes, RSS > 8.0 was an independent predictor of mortality and ischemic events at one year. Additionally, complete revascularization (RSS = 0) was associated with lower rates of adverse events [13].

In our study, we aimed to evaluate the relationship between WMR and RSS and the major adverse cardiac events (MACEs) developing in in-hospital and follow-up patients who underwent P-PCI due to STEMI.

## 2. Materials and Methods

**Figure 1:**
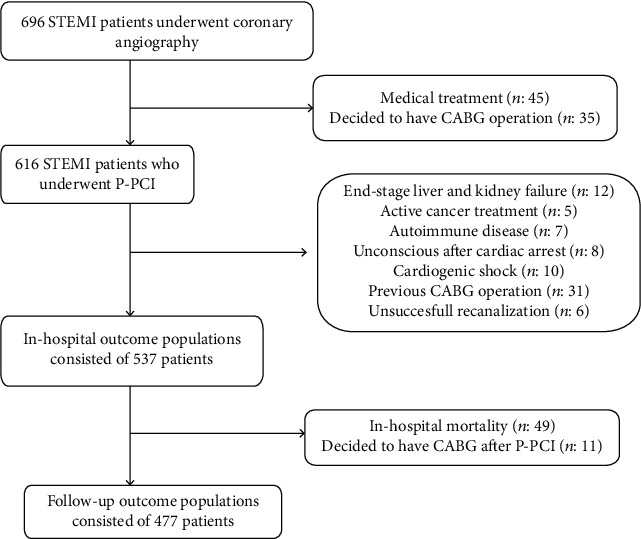
Flow diagram demonstrating enrollment and follow-up study patients.

This was a single-center, retrospective, observational cohort study that enrolled consecutive unselected non randomized eligible patients who were hospitalized for STEMI and underwent P-PCI at our institution from June 2015 to December 2018. A total of 696 patients were screened for involvement to study. Among these patients, 159 patients were excluded from the study, and the patients who were excluded from the study are presented in Figure 1. Finally, a total of 537 patients were included in the final study population. Clinical events of patients included the study, which developed in-hospital and after discharge until June 2019, were recorded, and retrospective analysis was performed. MACEs that developed in-hospital and after discharge were evaluated separately. Our local ethics committee approved the study protocol by the Declaration of Helsinki, and all patients provided written informed consent.

Demographic, clinical, and laboratory data at admission were collected from our hospital electronic database. Patients who had undergone PCI before and who had ≥50% stenosis in major coronary arteries and side branches, larger than 1.5 mm, on coronary angiography were defined as coronary artery disease. The diagnosis of STEMI was made according to current guidelines [14]. Biochemical and complete blood count (CBC) data were obtained from venous blood samples taken at admission. Blood samples were taken into standardized tubes containing ethylenedinitrilotetraacetic acid (EDTA). All measurements were processed 60 minutes on XE 5000 (Sysmex, Norderstedt, Germany). WMR was calculated using WBC and MPV values. Cardiac enzymes, creatinine levels, and hemogram parameters were studied daily during hospitalization. Peak troponin levels during hospitalization were evaluated in the study. Since troponin values above 50000 are given as >50000 in laboratory results, this situation was taken into consideration in our study. Contrast-induced nephropathy (CIN) was defined as a 25% increase in creatinine level compared to baseline or as an absolute increase of at least 0.5 mg/dl in the first 48 hours after P-PCI [15]. Major bleeding was defined according to the Bleeding Academic Research Consortium (BARC) definition (BARC type 3-5) [16]. GRACE score was calculated by using clinical, laboratory, and electrocardiographic data at admission [17]. All patients underwent echocardiography within 48-72 hours after P-PCI and after discharge in the first month. The modified Simpson method was used to calculate the left ventricular ejection fraction (LVEF).

### 2.1. Invasive Procedures

P-PCI was undertaken according to the European Society of Cardiology Guidelines and the operator's routine practice. The selection of the specific type of revascularization, procedural devices, stent types, and antiaggregant treatments were based on the decision of the operator. After the successful procedure, discharge medications were arranged in accordance with current STEMI guidelines [14].

The coronary arteries were classified based on anatomical criteria. The following vessels were considered as major coronary arteries: left main, left anterior descending, circumflex, and right coronary artery. Intermediate and isolated diagonal artery lesions were classified as other coronary vessels. MVD was defined as at least one lesion in a major noninfarct-related artery deemed angiographically significant (≥50% luminal narrowing diameter). Thrombolysis in myocardial infarction (TIMI) flow grade at the start and end of the procedure was determined from the angiographic films as previously described in previous studies [18]. Stent diameter refers to the maximal stent diameter; stent length is the sum of the length of all implanted stents. Multivessel PCI was defined as revascularization of at least one nonculprit lesion during the index procedure, before discharge or planned in the following 30-45 days without ischemic symptoms. Treatment of the nonculprit artery during P-PCI was left to the operator's preference. The decision to revascularize the nonculprit coronary artery after P-PCI was made by the heart team.

### 2.2. Baseline and Residual SYNTAX Score

Coronary angiograms were recorded to digital media for quantitative analysis. The SS and RSS were derived from the summation of the individual scorings for each lesion (defined as ≥50% stenosis in vessel ≥ 1.5 mm) on angiograms obtained before and after the procedure, respectively, as previously described [19]. All angiographic variables pertinent to SS and RSS calculations were computed by two of the experienced cardiologists who trained for SS assessment, and they were blinded to procedural data and clinical outcomes. In case of disagreement, the opinion of the third observer was obtained, and the final decision was made by consensus. All data were assessed for quality and entered into a dedicated computerized database. In our study, RSS score calculated after the procedure was evaluated in the results of the hospital, which was calculated after the intervention to infarct-related artery or after additional coronary vascular interventions outside infarct-related artery before discharge. If additional vascular intervention was not planned after discharge, the same RSS value was used for follow-up results. In patients who underwent additional vascular coronary intervention after discharge, the value of RSS calculated after PCI in the hospital was used for in-hospital results, and RSS calculated after follow-up additional PCI was used for follow-up results.

### 2.3. Clinical Endpoint Definitions

We classified endpoints into two groups as in-hospital and follow-up. Information about the in-hospital outcome was obtained from an electronic centralized clinical database. In-hospital clinical results were collected by a physician who was unaware of the initial clinical, laboratory, and angiographic results. In-hospital MACEs were defined as the combined endpoint of cardiac death, reinfarction, heart failure, and arrhythmic events. Cardiac death was defined as any death with a demonstrable cardiovascular cause or any death that was not attributable to a noncardiovascular cause. Reinfarction in acute post P-PCI phase was defined as clinical signs of reinfarction with recurrent or persistent symptoms and ST-segment changes requiring a repeat of P-PCI and/or second peak in CK-MB mass or troponin-T/ increase to ≥3 times the upper limit of normal, not related to an interventional procedure and new pathological Q waves in 2 or more contiguous electrocardiograph leads [20]. Heart failure was defined as the presence of signs or symptoms of congestion, mainly shortness of breath and signs of fluid retention. Arrhythmic events during hospitalization were defined as documented sustained ventricular tachycardia (VT) and ventricular fibrillation (VF). Safety endpoints were defined as combined endpoints of CIN and bleeding.

After discharge, at follow-up, clinical evaluation was made with 477 patients because 49 patients died in-hospital and 11 patients decided to undergo surgical treatment after P-PCI (Figure 1). All patients were contacted for the follow-up to assess the presence of MACEs. After discharge, all clinical follow-up data were prospectively collected by scheduled clinic evaluations, in-hospital records of the rehospitalized patients, and direct telephone interviews. In cases of unavailable information, we obtained information from the local citizen's registration office and medical charts. Follow-up clinical events were investigated and recorded by a physician unaware of the RSS score and in-hospital events.

MACEs of the follow-up period were defined as a combined endpoint consisting of cardiac death, reinfarction, heart failure, and revascularization. Cardiac death was defined as death caused by myocardial infarction, heart failure, sudden cardiac death, and cardiac procedures. Nonfatal reinfarction was defined as a new elevation of cardiac biomarkers associated with symptoms or new Q waves at 12-lead electrocardiogram or ST-T variation during symptoms [21]. Patients hospitalized with the diagnosis of decompensated heart failure were evaluated as the clinical endpoint of heart failure. Any repeat of revascularization was defined as any ischemia-driven target or nontarget vessel revascularization by either PCI or CABG [22].

### 2.4. Statistical Methods

Statistical analysis was made using IBM SPSS Statistics for Windows, Version 23.0 (IBM Corp., Armonk, NY). The normality assumptions were controlled by the Shapiro–Wilk test. Descriptive analyses were presented using mean ± SD, median (0.25–0.75 percentiles), or *n* (%), where appropriate. Categorical data were analyzed by Pearson chi-square and Fisher's exact tests. The differences between two groups were evaluated with Student's *t*-test for normally distributed data or Mann-Whitney *U* test for nonnormally distributed data. Spearman correlation coefficient was applied to find the correlation between continuous variables. Univariate and multivariate logistic regression analyses were used to determine independent risk factors associated with in-hospital MACE. To assess discriminative ability of the models, the C-statistic was calculated. The C-statistic is a unitless index of predictive discrimination, with a value of 0.5 indicating random prediction and a value of 1 indicating perfect prediction. The receiver operating characteristic (ROC) curve analysis was applied to evaluate the predictive performance of RSS, GRACE score, and WMR for MACEs and area under the curve (AUC); sensitivity and specificity were calculated and reported with 95% confidence intervals. The optimal cut-off point of measurements was determined as the value of the maximum Youden index. MACE curves were generated by the Kaplan-Meier method, and the log-rank test was used to evaluate differences between groups. Univariate and multivariate analyses of independent predictors of MACEs were performed with a Cox proportional hazard regression model. The variables which showed significant association with MACEs in the univariate analyses were further tested in the multivariate model. Hazard ratio (HR), with corresponding 95% confidence intervals (95% CIs), was reported. A *p* value of less than 0.05 was considered statistically significant.

## 3. Results

The median WMR value of 537 patients included in our study was 1286 (989-1645). Patients were divided into two groups as low (WMR < 1286, *n*: 268) and high (WMR ≥ 1286, *n*: 269) WMR groups were according to the median WMR value.

Baseline demographic, clinical, and laboratory data were analyzed and compared between the groups. Comparison results are given in Table 1. The high WMR group was older (*p* = 0.005) than the low WMR group and also the rate of smoking (*p* = 0.019) and hypertensive patients (*p* = 0.039) were higher. In clinical parameters, the high WMR group had a higher heart rate than the low WMR group (*p* = 0.017); patients with Killip > 2 heart failure (*p* = 0.009), intraaortic balloon pump support (*p* = 0.02), in need of intravenous inotropic (*p* = 0.01), intravenous diuretic (*p* = 0.04), and glycoprotein 2b3a therapy (*p* = 0.02) were higher than the low WMR group. In the high WMR group, systolic blood pressure (*p* < 0.001) and LVEF were lower than the low WMR group (*p* < 0.001). On admission (*p* = 0.003) and postprocedural 48-hour (*p* = 0.03) creatinine level and on admission CRP values (*p* < 0.001) were higher in the high WMR group. In the high WMR group, troponin-T levels and the rate of patients with peak troponin-T value > 50000 ng/ml were higher than those in the low WMR group (*p* < 0.001). Hematological parameters were analysed and WBC, neutrophil, PLT, and lymphocyte (*p* < 0.001) levels were higher in the high WMR group. MPV level of the high WMR group was lower than the low WMR group (*p* < 0.001).

Angiographic and procedural characteristics of patients included in the study are presented in Table 2. In the high WMR group, TIMI 0 flow rate before the P-PCI was higher than that in the low WMR group (*p* = 0.003) and TIMI 3 flow rate after the P-PCI was lower (*p* < 0.001). In the low WMR group, the rate of additional vessel intervention was higher than that in the high WMR group (*p* = 0.007). High WMR group's SS (*p* = 0.026) and RSS (*p* = 0.01) values were higher than those of the low WMR group.

In-hospital period, the high WMR group MACE rate was higher than that of the low WMR group (*p* = 0.001). Cardiac death (*p* < 0.001), decompansated heart failure (*p* = 0.007), and ventricular tachycardia/fibrillation rates (*p* = 0.003) were higher in the high WMR group than the low WMR group. Safety endpoints (*p* = 0.033) and CIN (*p* = 0.014) rates were higher in the high WMR group.

477 patients were evaluated for long-term cardiac events. Mean follow-up time of the study was 29 months (18-35), and there was no difference between the groups. In the follow-up period, MACEs (*p* = 0.043), cardiac death (*p* = 0.026), and reinfarction (*p* = 0.031) rates were higher in the high WMR group than in the low WMR group.  Table 3 shows the ratio of in-hospital and long-term MACEs.

The univariate and multivariate logistic regression model was performed to define the factors independently influencing in-hospital MACEs and the risk factors shown in Table 4(a). In Model 1 (with the GRACE score), independent predictors for in-hospital MACE were RSS, GRACE score, and LVEF, and in Model 2 (with the GRACE score and WMR), independent predictors were WMR, RSS, GRACE score, and LVEF. Models 1 and 2 displayed no lack of fitting (Hosmer-Lemeshow *p* value > 0.05). Based on the ROC curve analysis, the discrimination ability of Model 2 was better than that of Model 1 and it was significant (C‐statistics = 0.820 vs. 0.790 for Model 2 and Model 1, respectively, *p* = 0.038). Univariate and multivariate Cox proportional hazard models were performed to define the factors independently influencing follow-up MACEs, and risk factors and data are shown in Table 4(b). Independent predictors for long-term MACE were WMR and RSS.

**Figure 2:**
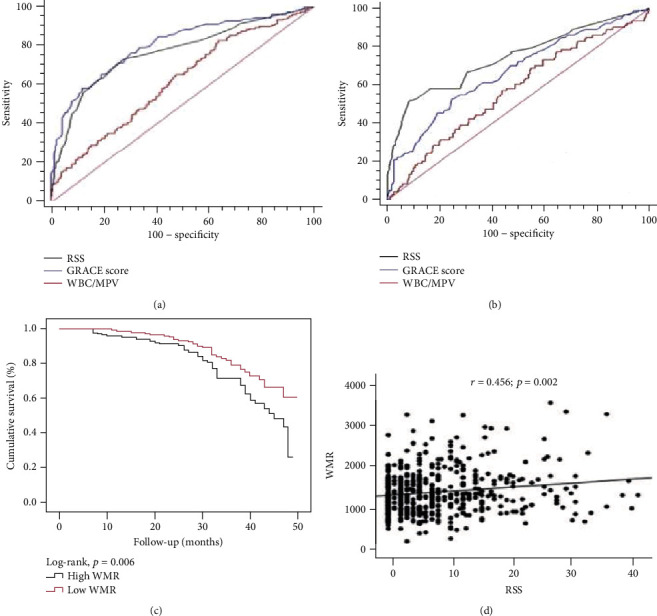
(a) ROC curve for in-hospital MACEs; (b) ROC curve for follow-up MACEs; (c) Kaplan-Meier survival curves for follow-up MACEs; (d) correlation between the WMR and RSS. MACE: major adverse cardiac event; RSS: residual SYNTAX score; WMR: white blood cell count to mean platelet volume.

ROC curve analysis was performed for cut-off values of WMR, RSS, and GRACE score, which predicted in-hospital and follow-up MACE (Figures 2(a) and 2(b)). Cut-off values for in-hospital MACE were found as >1064 (*p* < 0.001), >6 (*p* < 0.001), >121 (*p* < 0.001), respectively. AUC of WMR was lower than AUC of RSS (*p* < 0.001) and GRACE score (*p* < 0.001). Cut-off values for follow-up MACE were >1130 (*p* = 0.028), >11.5 (*p* < 0.001), and >119 (*p* < 0.001), respectively. AUC of WMR was lower than AUC of RSS (*p* < 0.028) and no different with GRACE score (*p* = 0.06). Comparative results of ROC analysis related to in-hospital and follow-up MACEs are presented in Table 5. The Kaplan-Meier analysis showed that a high WMR group had the lowest MACE-free survival rate (Figure 2(c)). Correlation analysis showed a moderate correlation between WMR and RSS (Figure 2(d)).

## 4. Discussion

In our study, there was a significant relationship between high WMR and in-hospital and follow-up MACEs in patients who underwent P-PCI due to STEMI. There was a relationship between in-hospital mortality, cardiac death, decompensated heart failure, ventricular tachycardia/fibrillation, CIN, and WMR. Follow-up MACEs, cardiac death, and reinfarction were higher in the high WMR group. A moderate correlation was observed between RSS and WMR. Regression and ROC analysis results showed that WMR can make an additional contribution for risk determination in STEMI patients.

WBC plays a vital role in progression of atherosclerosis and destabilization, and they can lead to thrombotic events. Increased WBC count is associated with increased mortality in STEMI patients [23]. In the study of Sabatine et al., the elevated WBC count was found to be a relevant mortality risk factor during the first 30 days and 6 months following the myocardial infarction among patients with ACS. In an analysis of 900 patients in the Stent Primary Angioplasty in Myocardial Infarction trial, investigators found that elevated WBC count upon hospital admission had a strong independent correlation with reinfarction and death at 1 year [24].

Inflammation, coronary thrombus load, platelet activation, and aggregation play an important role both in the pathogenesis of STEMI and in the development of adverse events. It has recently been observed that there is a close relation between cardiovascular mortality and the number of platelets or their ability to aggregate [25]. There is strong evidence indicating that MPV is an important variable and larger platelets have a higher thrombotic potential. MPV is a highly sensitive marker of platelet activity, and it could link the pathophysiology of diseases related to thrombosis and inflammation. The accumulated data suggest that MPV is a useful prognostic biomarker in patients with STEMI [26, 27].

MPV is expected to be increased in ACS patients. Increased MPV values are associated with MACEs and poor prognosis. In our study, in the high WMR group with high in-hospital mortality and MACE rates, high WBC rates were accompanied by low MPV levels. Similar to our study, there are studies showing that MPV levels may be low in ACS patients. Increased inflammation in ACS patients may have lowered MPV levels. Azab et al. demonstrated the relationship between decreased MPV values and increased inflammation in their study in the non-STEMI group. In a similar study, increased WBC count was accompanied by low MPV in ACS patients. This finding was following prior publications reporting an association of inflammation with low MPV [28, 29]. On the other hand, another study demonstrated that MPV decreased about 3 hours after the hospital admission and increased in the 3rd and 7th days in patients with STEMI and non-STEMI [30]. Changes in WBC and MPV levels due to increased inflammation and thrombogenicity may explain increased WMR levels in ACS patients.

WMR as a novel inflammation-based marker has recently been investigated as an independent predictor for long-term cardiovascular events in ACS patients [9]. Dehghani et al. reported that elevation in WMR levels at baseline was significantly associated with the incidence of MACEs in a long-term follow-up in patients with non-STEMI. WMR was stronger in predicting long-term MACEs than other complete blood cell indices in patients with non-STEMI [31]. Similarly, Çiçek et al. documented that higher admission WMR is to be a better predictor of long-term MACEs compared to such complete blood count indices as MPV, RDW, PLR, and NLR in patients with STEMI [32].

In our study, the highest WMR group consisted of high-risk patients according to demographic, clinical, laboratory, and angiographic findings. As a result of the current poor prognostic criteria, in-hospital mortality and MACE rates were higher in the high WMR group. In the follow-up period, rates of MACE, reinfarction, and cardiac death were higher in the high WMR group. Depending on the severity of the underlying inflammation during the follow-up period, existing coronary lesions may progress, de novo lesions may occur, and plaque rupture and ACS can be seen. This connection explains the relationship between the inflammatory marker WMR and follow-up MACEs. In particular, the relationship between reinfarction, cardiac death, and WMR shows that increased inflammation is associated with ACS in the follow-up period.

In ROC analysis, cut-off values of WMR were found in-hospital and follow-up MACE as 1064 (sensitivity: 83.12%, and specificity: 36.29%) and 1130 (sensitivity: 69.15%, and specificity: 44.91%), respectively. Although studies on ACS patients with different inflammation parameters have shown the relationship between the inflammation level and MACE, there are many factors that determine MACE in clinical practice. Comorbid factors, LVEF, medical treatment approaches, results, and complications of interventional treatments can affect treatment results and MACE regardless of the level of inflammation. Similarly to our study, due to the abovementioned adverse factors, in previous studies examining the relationship between inflammation parameters and MACE, the specificity rates are generally lower than the sensitivity rates [32, 33]. In risky clinical situations such as STEMI, markers that do not require additional costs, such as WMR, have a high sensitivity in determining risk and may be more valuable in clinical risk classification and treatment planning. Cut-off values in our study are compatible with previous studies; patients with WMR > 1000 may be accompanied by an increased risk of MACEs [34].

GRACE risk score is considered the most robust score for evaluating the risk in patients with STEMI/NSTEMI at initial presentation [17]. In our study, we investigated the possible contribution of the GRACE+WMR combination to the predictive effect of the GRACE score in the hospital MACE. Based on ROC curve analysis, the discrimination ability of GRACE+WMR (C‐statistics = 0.820) was better than the GRACE score (C‐statistics = 0.790). Evaluation of the GRACE score and WMR levels together in STEMI patients may provide more effective risk classification. Such risk stratification may allow clinicians to determine the therapy. More intensive medical and interventional treatment options can be considered in patients with high GRACE score and WMR.

SS has been recently developed as a combination of several previously validated angiographic classifications aimed at grading coronary anatomy concerning several lesions and their functional impact, location, and complexity. Scoring of lesions is weighed according to the size of the perfused territory of the left ventricle. SS has proven to be useful in risk stratification of non-STEMI and STEMI patients who underwent urgent PCI as an independent predictor of mortality and MACE [15]. In the study of Vogiatzis et al. in patients with ACS, MPV was significantly correlated to SS (*r* = 0.658, *p* < 0.001) and was found to be an independent predictor factor of MACEs [35]. Sivri et al. demonstrated the high SS association with increased WMR values in non-STEMI patients in their study [36]. Similarly, in our study, SS was found to be higher in the high WMR group. Increased WMR levels may be accompanied by complex and severe coronary artery lesions.

The RSS is defined as SS, remaining after completion of PCI, including cases of staged PCI procedure. Several observational studies with PCI patients with or without acute coronary syndrome using a cut-off either <8 or <5 for RSS found a significant reduction in MACE, death/MI/stroke, and unplanned revascularization procedures if the RSS after PCI was low [37]. In our study, for the first time, the relationship of WMR with MACEs in STEMI patients was evaluated with RSS, which is an indicator of residual ischemia. RSS was higher in high WMR values for in-hospital and follow-up periods. In addition to this analysis, RSS, LVEF, and GRACE score together with WMR were found as independent determinants of in-hospital MACE. Independent determinants of follow-up MACE were found as RSS and WMR. A moderate correlation was observed between RSS and WMR, and the time without MACE was lower in the highest WMR group. The relationship between RSS and WMR may explain the increase in MACEs as a result of ischemic events caused by the interaction of inflammation and residual coronary lesions.

WMR is obtained easily from routine blood counts without additional work or cost. According to the relevant analysis, the WMR can provide more effective risk stratification and additional prognostic information in addition to the clinical and anatomical parameters used for risk stratification of STEMI patients in daily practice.

### 4.1. Limitations

This was a retrospective observational and nonrandomized study conducted at a single hospital and as such has the inherent limitations and bias of retrospective single-center studies. Although the multivariable analysis was performed for significant confounders, we cannot exclude other potential unmeasured confounders that may affect the results. We did not interrogate any probable relationship between other complete blood count parameters and MACE and RSS of the patients. WMR was measured only once on admission; we could not evaluate the changes in WMR in response to aggressive treatment, due to lack of serial measurements. EDTA tubes were used for blood collection, and it has been shown that MPV increases with time when in contact with this anticoagulant. This limitation can be easily resolved as these measurements are performed in an automatic analyzer. Blood samples were measured within 60 min of sampling. We did not measure the markers of platelet-leukocyte interactions, including selectin molecules and adhesion ligands. However, our objective was to address the value of an easily available marker of platelet activation rather than more expensive and clinically unavailable options.

## 5. Conclusion

Our results illustrate that higher WMR value on admission was associated with worse outcomes in patients with STEMI and independently predictor for in-hospital and follow-up MACEs. WMR provides both a rapid and an easily obtainable parameter to reliably identify high-risk STEMI patients. Further large-scale studies are required to corroborate these results and establish the true role of the WMR clinical field.

## Figures and Tables

**Table 1:** In-hospital demographic, clinical, and laboratory characteristics of patients.

Parameters	All patients (*n*: 537)	Low (WMR < 1286) (*n*: 268)	High (WMR ≥ 1286) (*n*: 269)	*p* value
*Demographic parameters*				
Age (year)	61.88 ± 12.77	60.36 ± 12.78	63.42 ± 12.59	**0.005**
Male (*n*, %)	422(78.6)	206(76.9)	216(80.3)	0.332
*Clinical history*				
HT (*n*, %)	329(61.3)	136(58)	193(71.7)	**0.039**
DM (*n*, %)	255(47.5)	129(48.1)	126(46.8)	0.764
HL (*n*, %)	326(60.7)	164(61.2)	162(60.2)	0.818
CAD (*n*, %)	122(22.7)	63(23.5)	59(21.9)	0.663
Smoking (*n*, %)	217(40.4)	95(35.4)	122(45.4)	**0.019**
*Clinical parameters*				
Anterior MI (*n*, %)	256(47.7)	125(46.6)	131(48.7)	0.633
Inferior MI (*n*, %)	256(47.7)	128(47.8)	128(47.6)	0.967
Other localization MI (*n*, %)	25(4.7)	15(5.6)	10(3.7)	0.301
SBP (mmHg)	131.23 ± 28.21	135.63 ± 26.94	126.84 ± 28.81	**<0.001**
DBP (mmHg)	77.56 ± 16.37	78.25 ± 15.22	76.87 ± 17.45	0.329
Heart rate/min	82.52 ± 18.8	80.58 ± 16.91	84.45 ± 20.35	**0.017**
Killip > 2 HF (*n*, %)	102(19)	39(14.6)	63(23.4)	**0.009**
LVEF (%)	55(45-60)	60(48.5-63)	54(45-60)	**<0.001**
Inotropic treatment (*n*, %)	104(19.4)	37(13.8)	67(24.9)	**0.001**
IV diuretic treatment (*n*, %)	79(14.7)	31(11.6)	48(17.8)	**0.040**
Gp2b3a treatment (*n*, %)	86(16)	33(12.3)	53(19.7)	**0.020**
IABP (*n*, %)	23(4.3)	6(2.2)	17(6.3)	**0.020**
GRACE score	107(87-129)	106(87-126.5)	109(87-131)	0.484
Length of hospitalization (days)	5(4-5)	5(4-5)	5(4-5)	0.353
*Laboratory parameters*				
ABG level (mg/dl)	140(115-197)	138(112.5-198)	145(118-197)	0.299
FBG level (mg/dl)	108(92-151)	108(90-154)	109(92-148)	0.516
HbA1c	6.1(5.6-7.2)	6.1(5.6-7.3)	6(5.6-7.2)	0.701
A-Cre level (mg/dl)	0.89(0.76-1.03)	0.86(0.74-1)	0.9(0.8-1.09)	**0.003**
PP-Cre level (mg/dl)	0.91(0.8-1.1)	0.9(0.79-1.09)	0.93(0.8-1.2)	**0.030**
CreCl (ml/min)	87.26(69.28-99.01)	86.99(71.18-99)	88(68-99.4)	0.529
Peak trop-T (ng/ml)	50000(15.91-50000)	40.98(8.9-50000)	50000(24.65-50000)	**<0.001**
Peak trop-T(ng/ml) > 50000 (*n*, %)	282(52.5)	115(42.9)	167(62.1)	**<0.001**
CRP (mg/dl)	1.78(0.6-6.38)	1.38(0.48-4.07)	2.12(0.73-10.13)	**<0.001**
Hb (g/dl)	13.94 ± 1.95	13.8 ± 1.9	14.08 ± 1.98	0.090
WBC (×10^3^ *μ*/l)	10.91(8.63-13.5)	8.66(7.37-9.93)	13.3(11.49-15.3)	**<0.001**
Neutrophil (×10^3^ *μ*/l)	7.16(5-9.8)	5.32(4.13-7.05)	9.57(7.28-11.78)	**<0.001**
PLT (×10^3^ *μ*/l)	254(208-305)	228(191.5-275.5)	275(232-322)	**<0.001**
MPV (fl)	8.4(7.6-9.4)	8.8(8.1-9.9)	8(7.2-8.8)	**<0.001**
Lymphocyte (×10^3^)	2.31(1.55-3.3)	2.2(1.54-2.96)	2.49(1.56-4.07)	**0.001**
WMR	1286(989-1645)	989(844-1142)	1645(1450-1948)	**<0.001**

ABG: admission blood glucose; A-CRE: admission creatinine; CAD: coronary artery disease; CreCl: creatinine clearance; CRP: C-reactive protein; DBP: diastolic blood pressure; DM: diabetes mellitus; FBG: fasting blood glucose; Gp2b3a: glycoprotein 2b3a antagonist; Hb: hemoglobin; HbA1C: hemoglobin A1C; HF: heart failure; HL: hyperlipidemia; HT: hypertension; IABP: intraaortic balloon pump; IV: intravenous; LVEF: left ventricular ejection fraction; MI: myocardial infarction; MPV: mean platelet volume; PLT: platelet; PP-Cre: postprocedural creatinine; SBP: systolic blood pressure; trop: troponin; WBC: white blood cell; WMR: white blood cell count to mean platelet volume. Data are presented as mean ± SD, median (0.25–0.75 percentiles), and *n*(%). Student's *t*-test, Mann-Whitney *U* test, and Pearson chi-square test were used. Bold data are with statistical significance.

**Table 2:** In-hospital angiographic and procedural data characteristics of patients.

Parameters	All patients (*n*: 537)	Low WMR (*n*: 268)	High WMR (*n*: 269)	*p* value
One vessel (*n*, %)	156(29.1)	87(32.5)	69(25.7)	0.082
Multivessel disease (*n*, %)	381(70.9)	181(67.5)	200(74.3)	0.082
Three vessel disease (*n*, %)	163(30.4)	72(26.9)	91(33.8)	0.079
Two vessel disease (*n*, %)	220(41)	111(41.4)	109(40.5)	0.833
*Infarct-related artery*				
LAD (*n*, %)	247(46)	116(43.3)	131(48.7)	0.208
CX (*n*, %)	95(17.7)	54(20.1)	41(15.2)	0.136
RCA (*n*, %)	175(32.6)	87(32.5)	88(32.7)	0.951
LMCA (*n*, %)	7(1.3)	4(1.5)	3(1.1)	0.725
Other vessels (*n*, %)	13(2.4)	7(2.6)	6(2.2)	0.774
*TIMI flow grade*				
PrePro TIMI 0 (*n*, %)	295(54.9)	130(48.5)	165(61.3)	**0.003**
PrePro TIMI 1 (*n*, %)	26(4.8)	15(5.6)	11(4.1)	0.416
PrePro TIMI 2 (*n*, %)	38(7.1)	15(5.6)	23(8.6)	0.182
PrePro TIMI 3 (*n*, %)	178(33.1)	108(40.3)	70(26)	**<0.001**
PostPro TIMI 0 (*n*, %)	11(2)	4(1.5)	7(2.6)	0.364
PostPro TMI 1 (*n*, %)	16(3)	4(1.5)	12(4.5)	0.077
PostPro TIMI 2 (*n*, %)	34(6.3)	19(7.1)	15(5.6)	0.472
PostPro TIMI 3 (*n*, %)	476(88.6)	241(89.9)	235(87.4)	0.349
*Type of stent*				
BMS (*n*, %)	137(25.5)	69(25.7)	68(25.3)	0.901
DES (*n*, %)	400(74.5)	199(74.3)	201(74.7)	
*Number of stents*	1(1-1)	1(1-1)	1(1-1)	0.553
1 (*n*, %)	428(79.7)	211(78.7)	217(80.7)	0.577
>1 (*n*, %)	109(20.3)	57(21.3)	52(19.3)	
Stent length (mm)	22(16-30)	22(16-30)	24(18-30)	0.488
Stent diameter (mm)	3(2.75-3)	3(2.75-3)	3(2.75-3)	0.227
*Multivessel PCI (n, %)*	122(22.7)	74(27.6)	48(17.8)	**0.007**
Different time (*n*, %)	66(54.1)	39(52.7)	27(56.3)	0.701
LAD (*n*, %)	48(39.3)	32(43.2)	16(33.3)	0.274
CX (*n*, %)	51(41.8)	31(41.9)	20(41.7)	0.980
RCA (*n*, %)	40(32.8)	22(29.7)	18(37.5)	0.372
SS	17(10.5-24.5)	16(10-23.75)	18.5(12-24.5)	**0.026**
RSS	5(2-11.5)	4(0-10)	5(2-12)	**0.010**
Amount of contrast media (ml)	150(140-160)	160(150-170)	160(150-170)	0.807

BMS: bare metal stent; CX: circumflex; DES: drug eluting stent; LAD: left anterior descending; LMCA: left main coronary artery; PCI: percutaneous coronary intervention; NA: not applicable; PostPro: postprocedural; PrePro: preprocedural; RCA: right coronary artery; RSS: residual SYNTAX score; SS: SYNTAX score; TIMI: thrombolysis in myocardial infarction. Data are presented as median (0.25–0.75 percentiles) and *n*(%). Mann-Whitney *U* test, Pearson chi-square test, and Fisher's exact test were used. Bold data are with statistical significance.

**Table 3:** In-hospital and long-term MACEs according to WMR.

	All patients (*n*: 537)	Low WMR (*n*: 268)	High WMR (*n*: 269)	*p* value

*In-hospital endpoint*				
MACEs	154(28.7)	59(22)	95(35.3)	0.001
Cardiac death	49(9.1)	12(4.5)	37(13.8)	<0.001
Reinfarction	32(6)	11(4.1)	21(7.8)	0.070
DHF	107(19.9)	41(15.3)	66(24.5)	0.007
VT/VF	69(12.8)	23(8.6)	46(17.1)	0.003
*In-hospital safety endpoints*	95(17.7)	38(14.2)	57(21.2)	0.033
CIN	85(15.8)	32(11.9)	53(19.7)	0.014
Bleeding	15(2.8)	7(2.6)	8(3)	0.799

	All patients (*n*: 477)	Low WMR (*n*: 253)	High WMR (*n*: 224)	*p* value

*Long term endpoint*				
MACEs	94(19.7)	35(13.8)	59(26.3)	0.043
Cardiac death	26(5.5)	7(2.8)	19(8.5)	0.026
Reinfarction	29(6.1)	9(3.5)	20(8.9)	0.031
Heart failure	15(3.1)	8(3.2)	7(3.1)	0.982
Repeat revascularization	28(5.9)	13(5.8)	15(5.9)	0.954

CIN: contrast-induced nephropathy; DHF: decompensated heart failure; MACEs: major adverse cardiac events; RSS: residual SYNTAX score; WMR: white blood cell count to mean platelet volume; VT/VF: ventricular tachycardia/fibrillation. Data are presented as *n*(%). Pearson chi-square test was used.

**Table:** (a) Univariate and multivariate logistic regression analysis for determining the factors associated with in-hospital MACE

Variables	Univariate analysis		Multivariate analysis Model 1 (with GRACE)		Multivariate analysis Model 2 (with GRACE and WMR)	
	OR(95% CI)	*p*	OR(95% CI)	*p*	OR(95% CI)	*p*
Age	1.032(1.016-1.047)	**<0.001**	0.956(0.927-1.067)	0.103	0.960(0.930-1.017)	0.116
Male gender	0.852(0.544-1.333)	0.483	—	—	—	—
HT	1.584(1.065-2.356)	**0.023**	1.002(0.56-1.793)	0.993	0.996(0.556-1.785)	0.989
DM	1.79(1.226-2.613)	**0.003**	0.854(0.438-1.665)	0.644	0.847(0.434-1.653)	0.626
HL	1.571(1.058-2.332)	**0.025**	1.463(0.83-2.581)	0.189	1.493(0.844-2.642)	0.168
CAD	1.804(1.178-2.761)	**0.007**	1.275(0.699-2.326)	0.428	1.339(0.73-2.454)	0.345
Smoking	1.11(0.759-1.623)	0.590	—	—	—	—
Hba1c	1.284(1.156-1.426)	**<0.001**	1.129(0.937-1.361)	0.203	1.14(0.945-1.377)	0.171
CreCl (ml/min)	0.968(0.96-0.977)	**<0.001**	0.992(0.978-1.007)	0.295	0.995(0.98-1.009)	0.477
HB	0.85(0.772-0.936)	**0.001**	0.974(0.847-1.121)	0.715	0.947(0.821-1.092)	0.456
WBC × 10^3^	1.156(1.099-1.215)	**<0.001**	—	—	—	—
MPV	1.024(0.894-1.171)	0.736	—	—	—	—
WMR	2.805(1.752-4.488)	**<0.001**	—	—	1.764(1.046-2.976)	**0.033**
PLT	1.001(0.999-1.003)	0.331	—	—	—	—
LVEF	0.889(0.869-0.908)	**<0.001**	0.923(0.9-0.946)	**<0.001**	0.925(0.902-0.948)	**<0.001**
GRACE	1.045(1.036-1.054)	**<0.001**	1.043(1.027-1.059)	**<0.001**	1.042(1.026-1.058)	**<0.001**
RSS	1.145(1.112-1.179)	**<0.001**	1.070(1.033-1.107)	**<0.001**	1.067(1.03-1.105)	**<0.001**
C-Statistic (95% CI)	—	—	0.790(0.741-0.839)	**<0.001**	0.820(0.772-0.869)	**<0.001**
Hosmer-Lemeshow	—	—	5.143	0.424	8.102	0.742

**Table:** (b) Univariate and multivariate Cox regression analysis for determining the risk factors associated with follow-up MACE

Variables	Univariate analysis		Multivariate analysis	
	HR(95% CI)	*p*	HR(95% CI)	*p*
Age	1.039(1.021-1.057)	**<0.001**	1.017(0.993-1.041)	0.160
Male gender	0.683(0.438-1.066)	0.093	—	—
HT	1.098(0.716-1.685)	0.668	—	—
DM	1.659(1.1-2.501)	**0.016**	0.967(0.556-1.684)	0.906
HL	0.979(0.642-1.493)	0.921	—	—
CAD	1.32(0.84-2.075)	0.229	—	—
Smoking	0.56(0.363-0.862)	**0.009**	0.752(0.464-1.217)	0.245
Hba1c	1.154(1.035-1.288)	**0.010**	1.06(0.902-1.247)	0.479
CreCl	0.988(0.98-0.997)	**0.009**	1.006(0.994-1.018)	0.353
HB	0.973(0.889-1.065)	0.553	—	—
WBC	0.961(0.904-1.021)	0.197	—	—
PLT	1.002(0.999-1.004)	0.124	—	—
CRP (mg/dl)	1(1-1.001)	0.117	—	—
MPV	1.288(1.112-1.492)	**0.001**	1.153(0.969-1.372)	0.109
WMR	0.548(0.339-0.885)	**0.014**	0.775(0.478-1.256)	**0.01**
LVEF	0.966(0.949-0.983)	**<0.001**	0.988(0.968-1.009)	0.266
GRACE	1.019(1.012-1.026)	**<0.001**	1.006(0.996-1.017)	0.228
RSS	1.087(1.064-1.11)	**<0.001**	1.074(1.047-1.102)	**<0.001**

CAD: coronary artery disease; CreCl: creatinine clearance; CRP: C-reactive protein; DM: diabetes mellitus; Hb: hemoglobin; HbA1C: hemoglobin A1C; HL: hyperlipidemia; HT: hypertension; LVEF: left ventricular ejection fraction; MPV: mean platelet volume; PLT: platelet; RSS: residual SYNTAX score; WBC: white blood cell; WMR: white blood cell count to mean platelet volume. Bold data are with statistical significance.

**Table 5:** Cut-off values and comparison results of RSS, GRACE scores, and WMR related to in-hospital and follow-up MACEs.

	AUC	Cut-off value	Sensitivity (%)	Specificity (%)	*p* ^1^	*p* ^2^
*In-hospital MACE*					<0.001	<0.001
RSS	0.769(95% CI: 0.731-0.804; *p* < 0.001)	>6	73.38(95% CI: 65.7–80.2)	72.58(95% CI: 67.8–77)		
GRACE score	0.804(95% CI: 0.768-0.837; *p* < 0.001)	>121	65.58(95% CI: 57.5–73)	80.94(95% CI: 76.6–84.8)		
WMR	0.619(95% CI:0.576-0.660; *p* < 0.001)	>1064	83.12(95% CI: 76.2-88.7)	36.29(95% CI: 31.5–41.3)		
*Follow-up MACE*					0.038	0.06
RSS	0.744(95% CI: 0.702-0.0.783; *p* < 0.001)	>11.5	52.13(95% CI: 41.6-62.5)	91.38(95% CI: 88.1–94.0)		
GRACE	0.670(95% CI: 0.626-0.712; *p* < 0.001)	>119	53.19(95% CI: 42.6-63.6)	75.2(95% CI: 70.6–79.4)		
WMR	0.573(95% CI:0.528-0.618; *p* = 0.027)	>1130	69.15(95% CI: 58.8-78.3)	44.91(95% CI: 39.9–50.0)		

AUC: area under the curve; MACE: major adverse cardiac event; RSS: residual SYNTAX score; WMR: white blood cell count to mean platelet volume. *p*^1^: RSS versus WMR; *p*^2^: GRACE score versus WMR.

## Data Availability

The data used to support the findings of this study are included within the article. The data used to support the findings of this study can be obtained from the relevant author after the study is accepted to be published on demand.
